# Standard Hot Pressing as a Possible Solution to Obtain Dense K_0.5_Na_0.5_NbO_3_ Ceramic Doped by Er_2_O_3_ and Yb_2_O_3_

**DOI:** 10.3390/ma12244171

**Published:** 2019-12-12

**Authors:** Paweł Rutkowski, Jan Huebner, Adrian Graboś, Dariusz Kata, Dariusz Grzybek, Bogdan Sapiński

**Affiliations:** 1 Faculty of Materials Science and Ceramics, Department of Ceramics and Refractories, AGH University of Science and Technology, al. Mickiewicza 30, 30-059 Kraków, Poland; huebnerj@agh.edu.pl (J.H.); agrabos@agh.edu.pl (A.G.); kata@agh.edu.pl (D.K.); 2 Faculty of Mechanical Engineering and Robotics, Department of Process Control, AGH University of Science and Technology, al. Mickiewicza 30, 30-059 Kraków, Poland; dariusz.grzybek@agh.edu.pl (D.G.); deep@agh.edu.pl (B.S.)

**Keywords:** hot pressing, porosity, piezoelectric properties, sensors

## Abstract

In this study, the influence of the addition of rare earth oxides on the phase composition and density of KNN piezoelectric ceramics was investigated. The initial powders of Na_2_CO_3_ and K_2_CO_3_ were dried at 150 °C for 2 h. Then, a powder mixture for synthesis was prepared by adding a stoichiometric amount of Nb_2_O_5_ and 5 and 10 wt % overabundance of Na_2_CO_3_. All powders were mixed by ball-milling for 24 h and synthesized at 950 °C. The phase composition of the reaction bed was checked by means of X-ray diffraction (XRD). It had an appearance of tetragonal and monoclinic K_0.5_Na_0.5_NbO_3_ (KNN) phases. Then, 1 and 2 wt % of Er_2_O_3_ and Yb_2_O_3_, were added to the mixture. Green samples of 25 mm diameter and 3 mm thickness were prepared and sintered by hot pressing at 1000 °C for 2 h under 25 MPa pressure. The final samples were investigated via scanning electron microscopy (SEM)-energy-dispersive X-ray spectroscopy (EDS), XRD, Rietveld, and ultrasonic methods. Phase analysis showed tetragonal and orthorhombic KNN phases, and a contamination of (K_2_CO_3_·1.5H_2_O) was present. The obtained KNN polycrystals had a relative density above 95%. Texturing of the material was confirmed as a result of hot pressing.

## 1. Introduction

Piezoelectrics are seen as an important material group due to their place in a great variety of functional applications, ranging from simple sensors [1,2] to the interiors of the most advanced apparatuses, such as the atomic force microscope (AFM) [3]. In general, piezoelectricity stands for a reversible process of acquiring an electrical charge in solid material by applying mechanical stress. Materials that generate a measurably useful current are known as piezoelectrics and include various single crystals, organic materials, and perovskite ceramics.

In engineering and various industries, lead-based perovskites are predominantly used when piezoelectric effects are pursued. The most notable example of excellent electromechanical properties is witnessed in Pb(Zr,Ti)O_3_-based ceramics (PZT ceramics) [4]. Therefore, such materials have been widely studied and optimized for commercial and scientific usage, resulting in relatively simple processing methods and industry prevalence [5,6]. However, with constantly increasing awareness about health-endangering materials, the necessity of alternative, nonhazardous solutions has emerged. Compositions based on K_0.5_Na_0.5_NbO_3_ (KNN) are a possible alternative to replace lead-based piezoelectrics. Due to the significant piezoelectric response obtained by numerous teams [5,7,8,9], KNN-based materials have been widely researched, including in this study.

In a perovskite-type ABO_3_ structure, A-site cations are either alkaline earth elements or rare-earth elements, with B-site cations being transition metals. Theoretical structure consists of A cations occupying cuboctahedral coordination positions in the middle of eight corner-sharing BO_6_ octahedrons. Due to the specific structure and composition, perovskites have plenty of conductive properties used in material sciences, including significant piezoelectricity. The piezoelectric effect is directly derived from the noncentrosymmetric structure of materials. Such structures are electrically neutral, but lack symmetrical arrangement. Therefore, when mechanical stress is applied, and such structure shifts, it does not maintain a neutral charge [4]. The high magnitude of electromechanical effects obtained in perovskites is derived from the ordering tendency present in those materials, mainly the shift of B cations [4].

K_0.5_Na_0.5_NbO_3_ has a similar structure to BaTiO_3_, which consists of two phases, ferroelectric K_1-x_NbO_3_ and antiferroelectric Na_x_NbO_3_. Both phases have different ferroelectric transitions, crystallizing in a perovskite structure, with different symmetries. The phase transitions of KNN depend on the K/Na ratio [10]. To synthesize the basic structure of KNN, K, and Na carbonates are used in conjunction with Nb_2_O_5_ [5]:

$$K_{2}CO_{3}+Na_{2}CO_{3}+2Nb_{2}O_{5}\to 4K_{0.5}Na_{0.5}NbO_{3}+2CO_{2}^{↑}.$$

The main challenge emerging during KNN sintering is the densification of the obtained polycrystals. There are a few possible reasons that may cause this issue. During the thermal processing of KNN, evaporation of sodium is commonly reported [11,12]. Oxygen vacancies tend to appear during sintering processes [13]. Additionally, sintering of pure KNN is limited by lack of a liquid phase, resulting in a porous microstructure. Densification could be enhanced by the addition of various dopants. Rare-earth elements (REE) are reportedly used to address this issue due to the creation of a liquid phase and a high tolerance factor allowing for ion substitution in the perovskite structure [14].

REE oxides are used to significantly improve the piezoelectric properties of KNN-based materials. It was reported that the addition of these oxides resulted in a stabilizing and lowering dissipation factor in piezoelectric ceramics because of A-site ion substitution. The addition of two REEs was highlighted for the purpose of this study, ytterbium and erbium. Studies by Li et al. indicated that Yb^3+^ replaces both sodium and potassium (A site) and niobium (B site) in KNN structures, but each substitution can be promoted by changing the concentration of those cations. A low concentration (<0.25%) results in A-site substitutions, with Yb^3+^ cations acting as donor ions, while increased concentration (>0.25%) results in B-site substitutions, with Yb^3+^ cations acting as acceptor ions [15]. When a Yb^3+^ cation acts as a donor dopant, it causes the reduction of oxygen-vacancy concentration that appeared during sintering [16]. Elimination of vacancies is desired in that case to maintain the electrical resistance of KNN material. This phenomenon may lead to the desired deformation of a crystal structure [17]. The addition of Er_2_O_3_ has been investigated by Zhao et al. [18,19], and it enhances the photoluminescence properties of KNN ceramics. It also increases the coupling effects of mechanical-electrical luminescence. Er_2_O_3_ doping results in the appearance of a liquid phase during sintering, which is beneficial for material densification.

The aim of this study was to obtain a dense KNN-based material that could be used as a lead-free piezoelectric stress sensor. To achieve that two simultaneous approaches were investigated. First, 1 and 2 wt % amounts of Er_2_O_3_ and Yb_2_O_3_ as doping materials were proposed. Second, in order to increase the overall density of the material, uniaxial hot pressing in a graphite mold was employed instead of commonly used pressureless sintering. Such a process has a positive effect on texturing a polycrystal microstructure, which is desirable in this type of material due to the improvement of piezoelectric properties [20,21]. Additionally, it was expected that high pressure decreases sintering temperature, which, in turn, prevents alkali evaporation and reduces the effect of donor ions, increasing the KNN melting point. This allows to manufacture more stoichiometric K_0.5_Na_0.5_NbO_3_ material after thermal processing [22,23].

## 2. Materials and Methods

**Figure 1:**
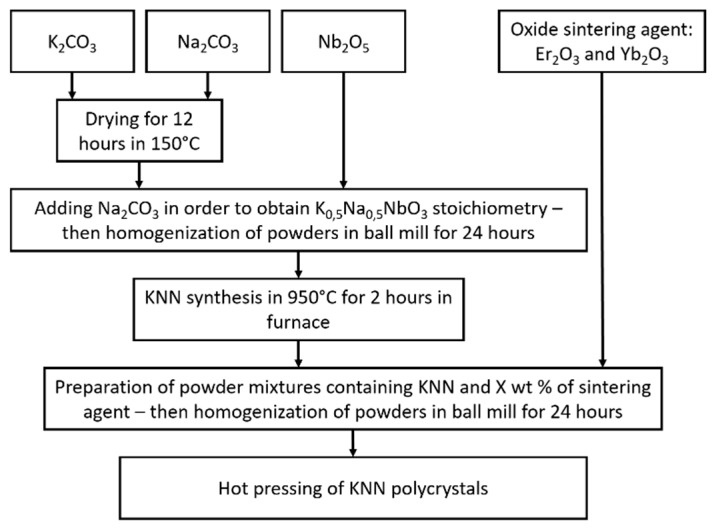
Procedure of K_0.5_Na_0.5_NbO_3_ (KNN) sample preparation.

Experiments were performed with commercially available powders of Nb_2_O_5_ produced by Changsha Easchem Co. Ltd. (Changsha, China, 99.7% purity); K_2_CO_3_ and Na_2_CO_3_ powders were produced by Lach-Ner (Neratovice, Czech Republic; 99.5% purity). In order to synthesize KNN, these powders were used as reactants for processing. To remove residual water from the samples, K_2_CO_3_ and Na_2_CO_3_ powders were dried at 150 °C for 12 4 and then kept in a desiccator between each step of the preparation process. Subsequently, K_2_CO_3_, Na_2_CO_3_, and Nb_2_O_5_ powders were initially mixed in ceramic mortar with an additional 5 and 10 wt % of Na_2_CO_3_ in order to avoid sodium deficiency due to evaporation during synthesis. The prepared powder mixture was then dry-homogenized in a ball mill for 24 h with low rotary speed. Silicon nitride milling media were used. Due to their high wear resistance, samples were unlikely to feature any contamination from this process. Next, the mixture was placed in a furnace inside an Al_2_O_3_ crucible at 950 °C for 2 h to complete KNN synthesis. Set amounts of Er_2_O_3_ and Yb_2_O_3_ sintering agents, shown in Table 1, were added to obtain KNN powder. Then, powders were additionally homogenized for 24 h in a rotary mill with the use of Si_3_N_4_ grinding media. Finally, circular green samples with a diameter of 25 mm and 3 mm thickness were prepared by hot pressing at 1000° C for 2 h of dwell time under 25 MPa pressure in a graphite mold in Ar gas flow. Due to differences in the atomic radius between K^+^ (0.133 nm) and Na^+^ (0.097 nm), sodium evaporates more easily during KNN sintering. A higher amount of K^+^ ions present in the KNN structure leads to a larger distance of the crystal face and results in a 2θ decrease of KNN patterns, which was confirmed by Jiang [24]. The procedure of KNN sample preparation is presented in Figure 1.

X-ray diffraction analysis was performed using a PANalytical X-ray diffractor (XRD, Almelo, The Netherlands) equipped with Cu tube, and X-pert HighScore software (version 3.0e) was used in order to designate sample phase composition. Scanning-electron-microscopy (NOVA NANO SEM 200, FEI EUROPE COMPANY, Czech Republic, Brno) observations with energy-dispersive X-ray spectroscopy (EDS of EDAX, Brno, Czech Republic,) analysis were performed using NOVA NANO SEM 200 equipped with an EDAX EDS analyzer (FEI, Brno, Czech Republic). Helium density of the prepared samples was measured using gas pycnometer AccuPyc II 1340 (Micrometrics, Norcross, GA, USA). Material texturing was examined by ultrasonic measurement using a CT-3 apparatus (Unipan Ultrasonic, Warsaw, Poland) equipped with a 1 MHz ultrasonic head.

## 3. Results

**Figure 2:**
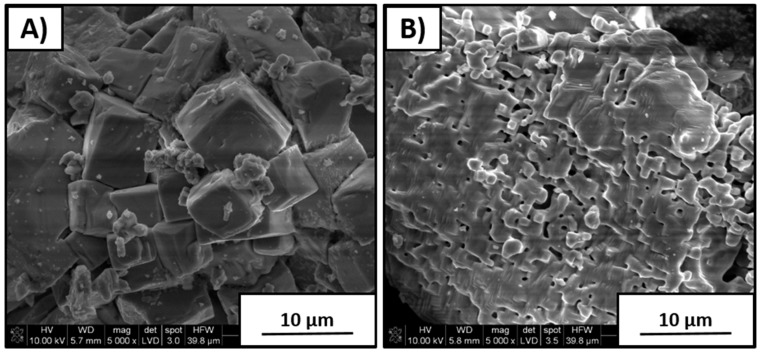
Scanning-electron-microscopy (SEM) images of products after synthesis with 1/1 K/Na molar reactants ratio: (**A**) coarse agglomerated particles and (**B**) partially sintered crystallites.

Figure 2 shows the overall morphological appearance of the products after the synthesis of the initial reactant mixtures of a stoichiometric K/Na molar ratio. Coarse particles with size in the range of 4–10 µm and partially sintered fine grains with dimensions of about 1 µm are visible in Figure 2A, B, respectively. In both cases, particles were strongly agglomerated. However, individual shapes of coarse grains are clearly distinguished. Particles shown in Figure 2B were five to eight times finer in comparison to those shown in Figure 2A.

**Figure 3:**
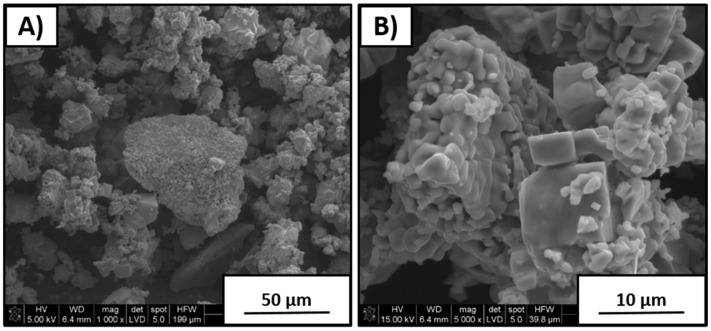
Powders with 5 wt % excess amount of Na_2_CO_3_ after synthesis. (**A**) 1000× magnification; (**B**) 5000× magnification.

Figure 3 shows SEM images of material synthesized with 5 wt % excess Na_2_CO_3_ amount. It can be seen that morphology was different than that for the sample with stoichiometric K/Na composition. Coarse particles with 8–10 µm size were covered by a thin layer of very fine crystallites with a size of 1–2 µm. Extensive formation of agglomerates was observed, as well as that an excess amount of Na_2_CO_3_ positively affected the nucleation of fine particles. Such finer grain-size distribution is expected to improve the overall density of polycrystals due to more efficient milling needed prior to sintering.

**Figure 4:**
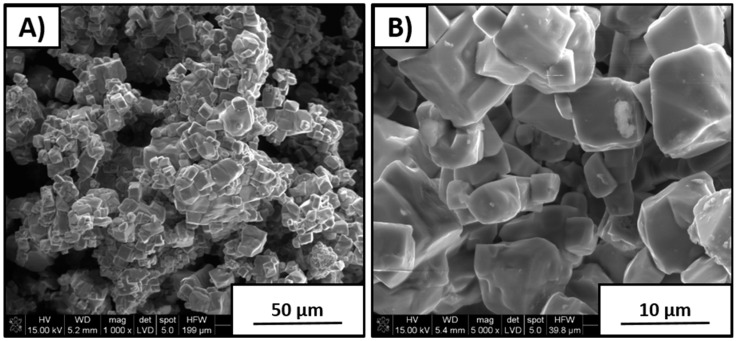
Powder morphology with 10 wt % excess amount of Na_2_CO_3_ after synthesis. (**A**) 1000× magnification; (**B**) 5000× magnification.

As seen in Figure 4, in the case of 10% sodium carbonate addition, powder morphology was significantly more uniform than that of the rest of the synthesized powders. It was characterized by a large amount of agglomerates formed from the cubical shapes of the particles. Deviation in size of the single crystallites varied I the range of 5–10 µm. Overabundance of 10 wt % Na_2_CO_3_ allowed for more homogeneous grain growth during KNN synthesis at 950 °C.

**Figure 5:**
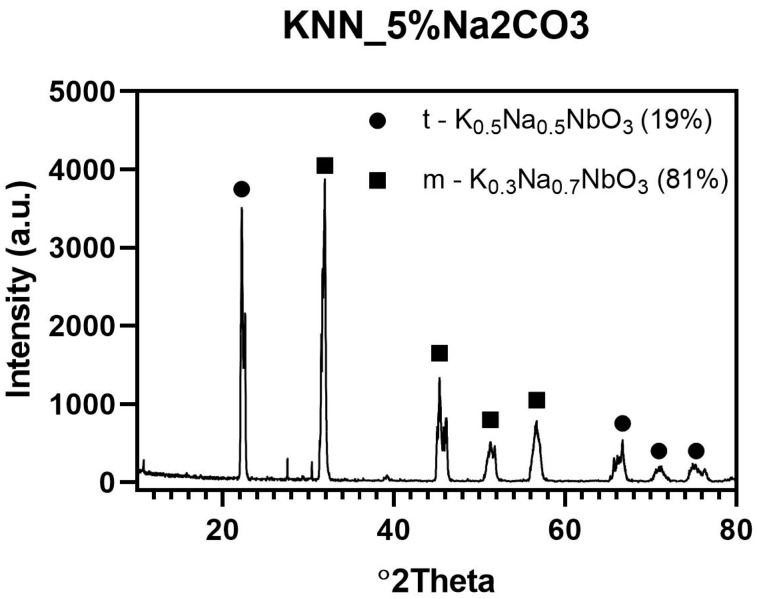
XRD pattern of sample synthesized with 5 wt % excess of Na_2_CO_3_ after 2 h thermal treatment at 950 °C.

Initial evaluation of the morphology of the synthesized KNN powders showed that the sample with 5 wt % overabundance of Na_2_CO_3_ was better suited for further processing. In order to check the phase composition of this powder, qualitative and quantitative XRD analysis was performed as shown in Figure 5. The presence of two different KNN phases was detected, tetragonal K_0.5_Na_0.5_NbO_3_ and monoclinic K_0.3_Na_0.7_NbO_3_. It was shown that synthesis conditions were correctly chosen to complete the reaction between reagents. After KNN synthesis, only about 19 wt % of tetragonal K_0.5_Na_0.5_NbO_3_ was obtained. Therefore, monoclinic K_0.3_Na_0.7_NbO_3_ (81%) are expected to transform into orthorhombic KNN phase after the sintering process with Er_2_O_3_ and Yb_2_O_3_ dopants, according to literature data [4].

**Figure 6:**
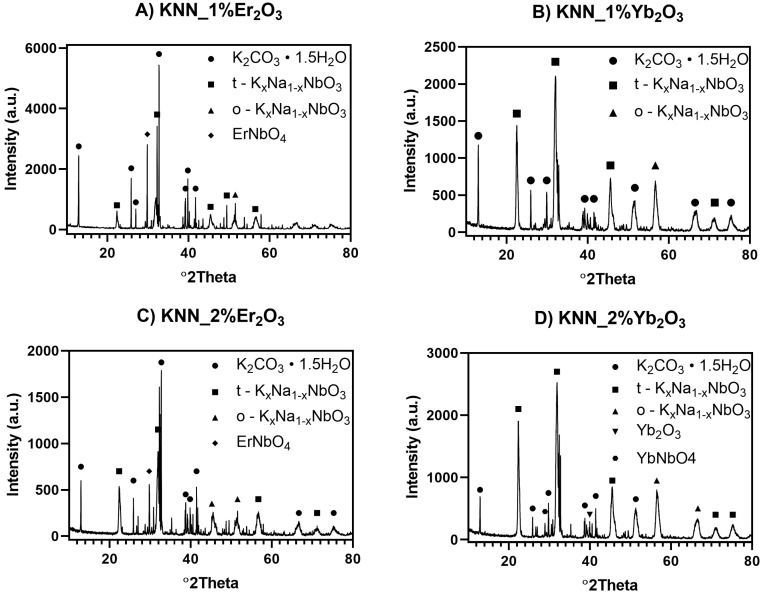
XRD patterns of hot-pressed samples with addition of rare-earth-element (REE) oxides as sintering agents and 5 wt % excess of Na_2_CO_3_. (**A**) KNN with 1% Er_2_O_3_ additive; (**B**) KNN with 1% Yb_2_O_3_ additive; (**C**) KNN with 2% Er_2_O_3_ additive; (**D**) KNN with 2% Yb_2_O_3_ additive.

The addition of a sintering agent in the form of rare metals oxides Er_2_O_3_ and Yb_2_O_3_ had a significant effect on the phase composition of the samples, as shown in Figure 6.

When qualitatively compared, the same phases were present in both samples, with an additional ErNbO_4_ and Er_2_O_3_ phase in the material doped by Er_2_O_3_. The quantity of erbium niobate increased and Er_2_O_3_ decreased with the addition of erbium oxide to the starting mixture. A similar increase in case of ytterbium niobate was seen in samples with ytterbium oxide introduced. However, quantitative analysis indicated that Yb_2_O_3_ substantially changed the sintering process in terms of acquiring tetragonal and orthorhombic KNN phases. Qualitative XRD phase-composition analysis showed that the synthetized powder obtained at 950 °C was composed of two KNN phases and contaminations, and it is presented in Table 2. A proportionally increasing amount of tetragonal and orthorhombic KNN was observed. The presence of dipotassium carbonate sesquihydrate (K_2_CO_3_·1.5H_2_O) was observed in each sample, which formed as the result of water absorption due to the hygroscopic properties of K_2_CO_3_. Water could be absorbed during extraction from the hot pressing (HP) apparatus or removal of the graphite foil layer used during the HP process, but the latter was performed in isopropanol to prevent such a reaction. K_2_CO_3_ appeared in the dense sintered material due to the reaction between the graphite form or graphite foil and the green KNN sample. XRD measurements were conducted on the material surface, and graphite was nonetheless not found in further EDS analysis of the fractured samples (Table 3), which may suggest that this effect occurred mainly on the material surface. The internal part of the sample was also free of water in comparison to the surface. Results indicated that Yb_2_O_3_ addition had a better effect on the final composition of the sintered samples than that of Er_2_O_3_. The overall amount of tetragonal and orthorhombic KNN phases was higher in samples containing Yb_2_O_3_. In Samples Y1 and Y2, the total weight percentage of the KNN phases exceeded 50% of the material. The appearance of orthorhombic KNN was the result of the phase transformation of the monoclinic phase detected in the powder after synthesis at 950 °C.

**Figure 7:**
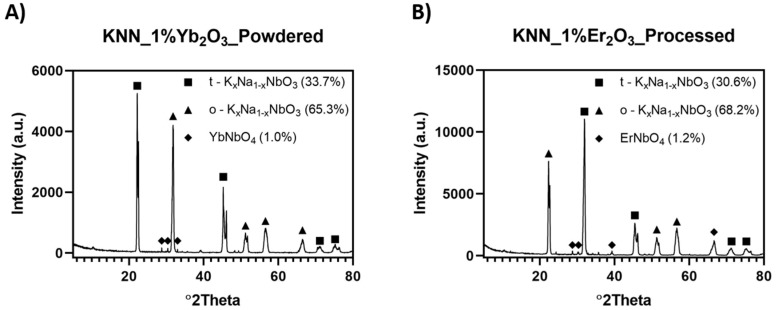
XRD analysis of (**A**) pulverized KNN +1% Yb_2_O_3_ and (**B**) KNN + 1% Er_2_O_3_ with mechanically removed thin surface layer.

The existence of K_2_CO_3_ in the hot-pressed materials was also examined in the volume of the material by two methods. First, the selected material was gently pulverized in a ceramic SiC mortar, followed by XRD analysis, which is shown in Figure 7A. Second, the surface layer of KNN + 1% Er_2_O_3_ was mechanically removed, and the result is illustrated in Figure 7B. This confirmed that K_2_CO_3_ was not present in the sample volume. Phase analysis showed the existence of tetragonal and orthorhombic KNN with the addition of ErNbO_4_ or YbNbO_4_. This indicated that K_2_CO_3_ only formed on the material surface.

**Figure 8:**
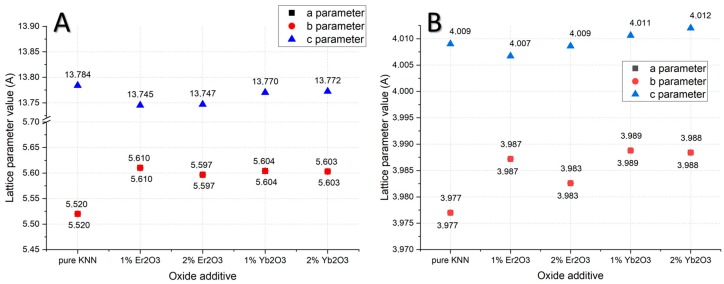
Lattice-parameter changes versus rare-earth oxide addition for (**A**) o-KNN and (**B**) t-KNN (a and b parameters overlapped).

XRD data analysis with a comparison to the reference material showed differences in the lattice-parameter values of KNN structures induced by erbium and ytterbium dopants (Figure 7). The lattice parameters of pure orthorhombic KNN were a,b = 5.5200 Å, and c = 13.7840 Å. In the case of 1% Yb_2_O_3_ addition, they were a,b = 5.6042 Å, and c = 13.7703 Å. For 2% Yb_2_O_3_ addition, they were a, b = 5.6031 Å, and c = 13.7727 Å. Therefore, lattice-structure deformation was visible in the shrinkage of the “a” and “b” parameters, and in the elongation of the “c” parameter. A similar situation was observed in erbium doped samples with a visibly higher shrinkage of the “c” parameter (Figure 8A). In case of a tetragonal structure, the “c” parameter was elongated with the addition of Yb_2_O_3_, differing from samples in the orthorhombic structure (Figure 8B). In both structures, higher additions of dopants led to a decrease of the “a” lattice parameter, which agreed with data from the literature [15].

**Figure 9:**
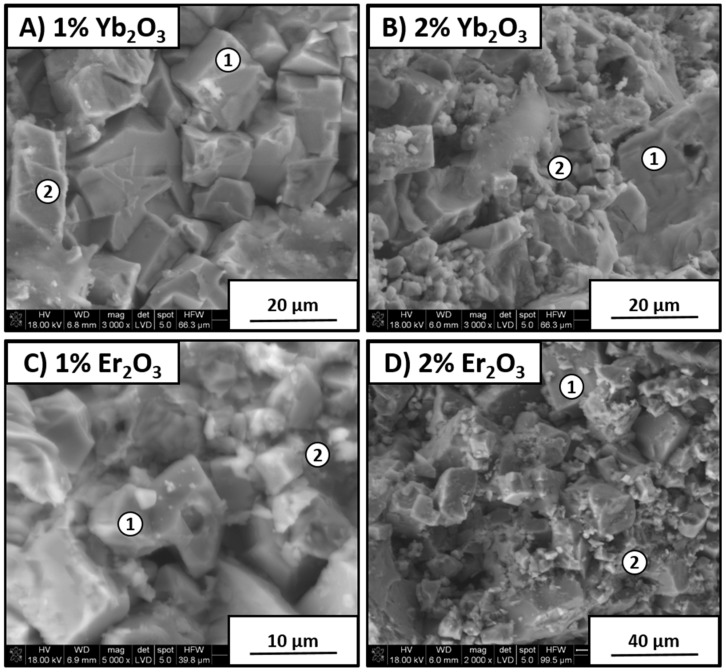
SEM sample images with different sintering agents and energy-dispersive X-ray spectroscopy (EDS point analysis marked on images): (**A**) 1% Y_2_O_3_, (**B**) 2% Y_2_O_3_, (**C**) 1% Er_2_O_3_, and (**D**) 2% Er_2_O_3_.

Figure 9 shows samples with 1 and 2 wt % addition of Er_2_O_3_ and Yb_2_O_3_ sintering agents. It can be seen that both types of material had similar morphology. In the case of samples containing 1 wt % oxide additions, grains were bigger than those for materials with 2 wt % additions. This indicates that more of the sintering agent caused fine KNN grains to appear. This was confirmed by EDS analysis, as shown in Table 3.

The sample density is presented in Table 4. All prepared materials were characterized by high relative density above 95%, with the highest of 99.4% in the addition of 2% Er_2_O_3_. The 5 wt % excess of Na_2_CO_3_ caused non-uniform grain-size distribution after the synthesis process. The addition of rare-earth sintering agents of Er_2_O_3_ and Yb_2_O_3_ with high pressure and temperature during hot pressing allowed good powder compaction, which led to obtaining dense polycrystals.

**Figure 10:**
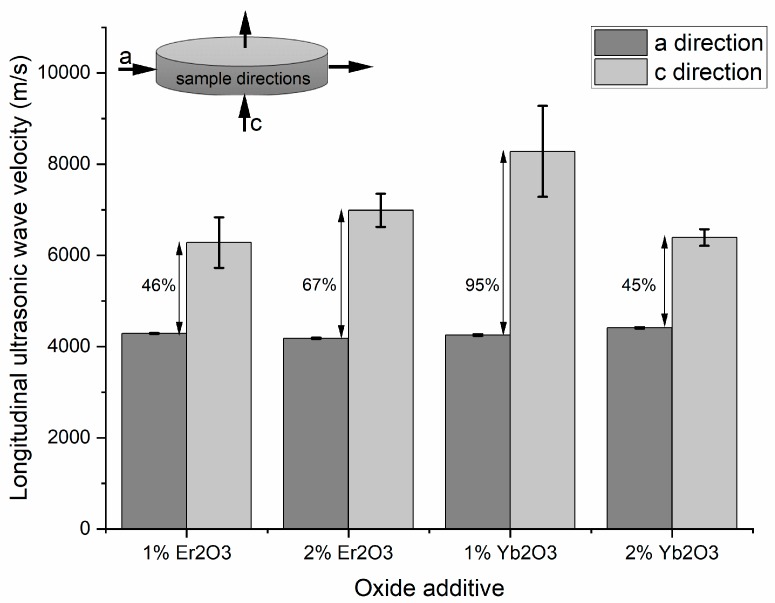
Ultrasonic-wave-velocity examination in parallel and perpendicular direction to the hot pressing (HP) axis.

KNN polycrystal analysis revealed the anisotropic feature of their microstructure. Ultrasonic measurements were conducted in two different directions on each sample, as indicated in Figure 10. The difference in longitudinal ultrasonic-wave velocity measured in the “a” and “c” directions was higher than 45% and reached almost 95%. Wave velocities differed significantly for each sample, so texturing of the material as an effect of applied pressure during the hot-pressing process was observed.

## 4. Discussion

In order to prepare high-quality KNN powder after synthesis, overabundance of Na_2_CO_3_ was necessary because of the expected evaporation of sodium during thermal processing. The morphology of KNN powders made from reactants after synthesis showed that material containing 5 wt % excess of Na_2_CO_3_ allowed the formation of coarse and fine particles due to the presence of fine grains; the appearance of liquid phase was expected that could lead to improved density after sintering. XRD analysis of the 5 wt % Na_2_CO_3_ synthesized powder showed the presence of two phases: tetragonal K_0.5_Na_0.5_NbO_3_ and monoclinic K_0.3_Na_0.7_NbO_3_. After homogenization with Er_2_O_3_ and Yb_2_O_3_ dopants, and subsequent hot pressing, the monoclinic phase transitioned into orthorhombic K_0.5_N_0.5_NbO_3_. However, contamination of K_2_CO_3_·1.5H_2_O was detected on the surface of all materials due to a reaction with the graphite mold and exposure to the atmosphere. The addition of Er_2_O_3_ resulted in the appearance of small amount of ErNbO_4_ in the final samples. The formation of ErNbO_4_ during hot pressing reduced the porosity of the samples. Ultrasonic-wave-velocity examination in the parallel and perpendicular direction to the HP axis indicated the texturing of the sintered material.

Results showed that hot pressing was an efficient method to obtain dense KNN polycrystals. However, to avoid unwanted carbon diffusion to the sintered material, nonreactive isolation should be applied during sintering. There are two possible solutions: first, change the mold material from graphite to alumina. Second, including an additional protective cover between graphite and the inner material layer.

## 5. Conclusions

- Synthesis of a powder containing 5 wt % excess of Na_2_CO_3_ resulted in the appearance of fine crystals and coarse grains, beneficial for densification during sintering due to the appearance of liquid.
- Dipotassium carbonate sesquihydrate (K_2_CO_3_·1.5H_2_O) appeared at the surface of the material due to the reaction with carbon derived from the graphite mold and later exposure to the atmosphere. The thin carbonate layer could be mechanically removed.
- The addition of sintering agents resulted in the transformation of the monoclinic phase into orthorhombic KNN in the final samples.
- Hot pressing allowed us to obtain samples with relative density over 95%.
- It was confirmed that hot pressing was beneficial for KNN texturing after sintering.

## Figures and Tables

**Table 1:** Sample composition.

Sample ID	K_0,5_Na_0,5_NbO_3_ (wt %)	Er_2_O_3_ (wt %)	Yb_2_O_3_ (wt %)
Reference sample	100	-	-
E1	99	1	-
E2	98	2	-
Y1	99	-	1
Y2	98	-	2

**Table 2:** Composition of sintered samples in weight percentage.

Sample ID	Figure No	K_2_CO_3_•1.5H_2_O (%)	t-KNN (%)	o-KNN (%)	ErNbO_4_ (%)	Er_2_O_3_ (%)	YbNbO_4_ (%)	Yb_2_O_3_ (%)
E1	6A	67.4	23.0	8.0	0.7	1.0	-	-
E2	6C	61.9	28.0	8.6	1.1	0.4	-	-
Y1	6B	47.1	35.2	16.7	-	-	0.4	0.6
Y2	6D	33.9	44.0	20.3	-	-	1.2	0.6

**Table 3:** SEM EDS weight percentage for samples shown in Figure 8.

Sample and Point	Figure No.	K (%)	Na (%)	Nb (%)	O (%)	Yb (%)	Er (%)
Y1-1	8A	13	6	58	19	4	-
Y1-2	8A	12	6	57	21	4	-
Y2-1	8B	15	5	57	20	3	-
Y2-2	8B	8	9	56	23	4	-
E1-1	8C	16	4	61	14	-	5
E1-2	8C	15	4	63	14	-	4
E2-1	8D	12	3	68	11	-	6
E2-2	8D	10	8	53	26	-	3

**Table 4:** Density of obtained samples.

Sample ID	NaCO_3_ Excess (wt %)	Sintering Aid	Sintering-Aid Amount (wt %)	Hot Pressing Temperature (°C)	Helium Density (g/cm^3^)	Relative Density (%)
E1	5	Er_2_O_3_	1	1000	4.40 ± 0.02	96.8
E2	5	Er_2_O_3_	2	1000	4.54 ± 0.02	99.4
Y1	5	Yb_2_O_3_	1	1000	4.34 ± 0.04	95.6
Y2	5	Yb_2_O_3_	2	1000	4.45 ± 0.03	97.8
